# Analysis of the Role of Selective Neck Dissection in Clinically Node-Positive T3/T4 Oral Cancers

**DOI:** 10.1155/2022/2204745

**Published:** 2022-02-10

**Authors:** Dungala Dileep Maharaj, Rajkumar Kottayasamy Seenivasagam, Kinjal Shankar Majumdar, Abhinav Thaduri, Achyuth Panuganti, Pallvi Kaul, Jarang Rajesh Kumar, Nooruddin Mohammed

**Affiliations:** ^1^Department of Otorhinolaryngology and Head and Neck Surgery, All India Institute of Medical Sciences, Rishikesh, India; ^2^Department of Surgical Oncology, All India Institute of Medical Sciences, Rishikesh, India; ^3^Department of General Surgery, All India Institute of Medical Sciences, Mangalagiri, India; ^4^Department of General Surgery, Dr. Pinnamaneni Siddhartha Institute of Medical Sciences and Research Foundation, Vijayawada, India

## Abstract

**Introduction:**

The concept of selective neck dissection (SND) in locally advanced oral cancers is emerging. Contemporary studies support the feasibility of SND in selected node-positive oral cancers with early primaries. Nevertheless, the suitability of SND in clinically node-positive (cN+) oral cancers with advanced primaries (T3/T4) is unknown.

**Aim:**

This study explores if patients with cN+ advanced primaries were suitable candidates for SND by spotting the involved lymph node distribution in various stations of the neck. Secondary objectives were to check if predictive clinicopathological factors for metastases to the neck in general also apply for lymph node metastases to levels IV and V.

**Methods:**

The present retrospective study analysed the distribution of pathologically involved lymph nodes in 134 patients and explored the interrelation of various predictive factors and cervical metastases overall and those specific to levels IV and V.

**Results:**

Level V was involved in 6.7% (6/83) of T4 and none of the T3 primaries. Depth of invasion (DOI), perineural invasion (PNI), and skin invasion were statistically significant predictors for nodal metastases in general on multivariate analysis.

**Conclusion:**

Our analysis supports the option of considering SND, sparing level V in patients with cN+ oral cancers in a subset with T3 primary and nodal stage N2 and below.

## 1. Introduction

Oral cancer (OC) burden has a variable distribution across the globe owing to variable regional, ethnic, and socioeconomic differences and variations in the prevalence and severity of risk factors in these communities, including oral smokeless tobacco in Southeast Asia. Recent GLOBOCAN (Global Cancer Observatory) statistics estimated 377,713 new oral cancer cases with 177,757 deaths throughout the world in 2020 [1]. Current treatment for locally advanced oral cancers is a multimodality approach comprising surgery and chemoradiotherapy. Metastases to regional lymph nodes are associated with poor prognosis and decreased overall survival. Management of the neck in oral cancers during surgery includes selective neck dissection (SND) in node-negative patients and some type of comprehensive neck dissection (CND) in node-positive patients [2].

However, as nodal metastases to levels IV and V are rare in the absence of involvement of upper levels, an increasing number of studies have shown evidence for feasibility of SND even in some node-positive oral cancers [3]. The meta-analysis by Liang et al. [4] showed no difference in regional recurrence, disease-specific survival, or overall survival for patients treated with CND or SND in patients with clinically node-positive (cN+) oral cancers.

Again, however, the majority of studies assessing the practicability of SND in clinically node-positive neck (cN+) have been done in patients with early “T stage” cancers or cancers with a single node in the higher neck levels [5–13]. Whether SND is expedient in cN+ oral cancers with advanced primaries is still an unexplored area to the best of our knowledge.

Besides, literature review revealed that close to half of the studies supporting SND arrived at their conclusions considering all head and neck cancers as a single cohort [6, 8, 10]. Since not all subsites of head and neck cancers follow the same pattern of metastasis, conclusions derived may not be uniformly applicable. Moreover, chances of neck nodal metastases increase with the size of the primary lesion: early and advanced oral cancers do not behave in the same way with respect to neck nodal spread [5].

A meta-analysis by Liang et al. [4] showed that SND with adjuvant radiotherapy in positive node patients showed no significant differences in regional control, overall survival, or disease-specific survival compared to comprehensive neck dissection in oral cancer patients. The systematic review by Rodrigo et al. [14] showed the feasibility of SND in cN1 and selected cN2 cases. One major drawback was that they considered all head and neck cancers together for analysis. In addition, they did not analyse tumor factors (stage) that influence the extent of neck dissection required.

Against this background, we wanted to explore if any subset of advanced oral cancer (T3/T4) patients with cN+ were suitable candidates for SND by retrospectively analysing the pathological lymph node distribution in the neck and identify any preoperative clinical, pathological, and demographic factor(s) that can predict metastases to levels IV and V. This would provide us data to design a prospective clinical trial on such patients.

## 2. Methodology

This retrospective analysis was done at the All India Institute of Medical Sciences, Rishikesh, India. Data was retrieved from medical records of oral cancer patients operated between August 2018 and November 2020. Institute ethics committee approval including waiver of individual patient consent was obtained. All oral cancer patients (oral squamous cell carcinoma) with advanced primaries (cT3/T4) and clinically node-positive neck (cN+) who had undergone curative surgery including for the neck were included for analysis. Patients with prior treatment (surgery/radiotherapy/chemotherapy), nonsquamous cancers, salvage/recurrent surgeries, and patients with missing details were excluded from the study. Patients were staged as per the AJCC 8th edition staging system. During surgery, the neck was addressed with ipsilateral CND or contralateral CND/SND when disease was reaching or crossing the midline.

### 2.1. Statistical Analysis

For ease of comparison, continuous data were converted into categorical groups. All categorical variables were expressed as frequencies with percentages (%). A chi-square test was used to check the association between nominal variables. Strength of association was measured with the phi coefficient for dichotomous variables and Cramer's V for polychotomous variables. To look for the cause-effect association of variables exhibiting significant association, binomial and multinomial logistic regression analysis was applied. Statistical analysis was done with SPSS Statistics V26.0 (IBM Corp, Armonk, NY, USA), and a *p* value of less than 0.05 was considered significant.

## 3. Results

### 3.1. Clinicopathological Variables

The majority of the patients were males (*n* = 119, 88.8%), and 83.6% were 60 years or older. Smokeless tobacco was the most common form of addiction (*n* = 99, 73.9%), and more than half of the population had multiple comorbidities (CCI score > 2, *n* = 72, 53.7%) and multiple addictions (*n* = 74, 55.2%). Buccal mucosa (*n* = 70, 52.2%) was the most common subsite involved, followed by the tongue (*n* = 29, 21.6%) and lower alveolus (*n* = 23, 17.2%). Disease was crossing the midline in 24.4% (*n* = 33) of patients. Clinically, 35 (26.1%) patients were staged as N1, 77 (57.5%) patients as N2, and the remaining 22 ( 16.4%) were staged as N3. Pathological node positivity (pN+) was seen in 83 (61.9%) patients. Levels Ib (86, 34.6%) and IIa (84, 33.8%) were frequently involved levels followed by level III (35, 14.1%). Only 5.2% of involved lymph nodes were in levels IV and V. Contralateral neck dissection was done in 31 patients. Contralateral lymph node positivity was seen in 8 patients (level Ib in 4 patients, IIa in 3 patients, and both Ib and IIa in one patient).

Two-thirds of patients (*n* = 89, 66.4%) had T4 disease. Poor pathological prognostic factors like depth of invasion (DOI) > 10 mm, perineural invasion (PNI), lymphovascular invasion (LVI), and extranodal extension (ENE) were present in 86.6% (*n* = 116), 17.2% (*n* = 23), 13.4% (*n* = 18), and 16.4% (*n* = 22) of the cohort, respectively. Pathological bone involvement was 34.3% (*n* = 46), and skin involvement was 41.8% (*n* = 56). Baseline clinical and pathological parameters are shown in Table 1.

### 3.2. Pathological Lymph Node Distribution by T Stage

There was no significant difference in pathological node involvement between T3 (66.7%) and T4 (59.6%) tumors. Most common lymph node stations that were involved were level Ib (T3-46.7%, T4-39.3%) and level IIa (T3-44.4%, T4-37.1%). Level Ia (T3-11.1%, T4-16.9%) and level III (T3-15.6%, T4-16.9%) were the next frequently involved stations. Level IV was involved in only 2.2% of the cases of both T3 and T4. All cases with level V nodal involvement (6.7%) were T4 tumors (Table 2).

### 3.3. Pathological Lymph Node Distribution by N Stage

Lymph node involvement was confined to stations I and II in patients with N1 disease (Ia-1.5%, Ib-6.7%, IIa-8.2%, IIb-0.7%). Level III, IV, and V involvement was noted in advanced nodal stages (N2 and N3). Even in higher N stages, most of the involved lymph nodes were located at level Ib (N2-23%, N3-11.9%), IIa (N2-19.4%, N3-11.9%), and III (N2-6%, N3-10.4%). Level IV was involved in N2 (1.5%) and N3 (0.7%). Level V lymph node positivity was most prevalent in the N3 subgroup (3%) (Table 3).

### 3.4. Subsite-Wise Distribution of the Pathologically Involved Lymph Nodes

We also analysed distribution of pathologically involved lymph nodes as per subsite. Nodal metastases were found in 75.9% (22/29) of tongue, 69.6% (16/23) of lower alveolus, and 52.9% (37/70) of buccal mucosa cancers. Levels Ib and IIa were the most commonly involved nodal levels. Level IV metastases (3/3) were seen only in the tongue subsite, while level V metastases (4/6) were predominantly associated with the lower alveolus.

### 3.5. Association between Clinicopathological Factors and Nodal Metastases

We analysed the association between various clinicopathological parameters and on nodal metastases, including level IV and V metastases in particular using regression analysis (Table 4).

Factors exhibiting cause-effect association with overall nodal metastases on univariate analysis were skin excision during primary tumor resection (*p* = 0.05), DOI > 10 mm (*p* = 0.01), PNI (*p* = 0.006), LVI (*p* = 0.02), and pathological skin invasion (*p* = 0.001). ENE at other levels was the only factor manifesting statistical significance for metastases to levels IV (*p* = 0.006) and V (*p* = 0.05). Majority of cases with level IV metastases had primaries crossing the midline, and this association was significant (*p* = 0.002). Alcohol consumption (*p* = 0.08) was showing a trend towards significance for level V metastases. On multivariate analyses, predictive factors for nodal metastases were DOI > 10 mm (*p* = 0.01, OR: 4.6, 95% CI: 1.40-15.5) and skin invasion (*p* = 0.005, OR: 5.9, 95% CI: 1.72-20.25).

## 4. Discussion

A study on the distribution of regional metastasis by Shah et al. in 1990 laid the foundation for studies to look for the possibility of SND in oral cancers. Their analysis showed that 96.5% of patients had involved lymph nodes located in levels I, II, or III [15].

For oral cancers, proper management of lymph node basins in the neck is of priority. Substantial evidence is available supporting selective neck dissection rather than observation in node-negative oral cancers [16]. Simultaneously, studies are emerging to limit neck dissection in node-positive oral cancers [4]. Quoted reasons for the same are functional and cosmetic morbidity associated with comprehensive neck dissections, especially shoulder dysfunction [2, 15, 17].

Kowalski et al. showed ipsilateral lymph node metastases in >50% of T3 and T4 oral cavity lesions [5]. Our survey revealed pathological node positivity only in 61.9% (83/134) patients, though all were clinically positive for palpable nodes. All patients had clinically palpable lymph nodes limited to levels I/II/III. None had lymph nodes in levels IV or V. But the distribution of pathologically positive lymph nodes stretched from levels I to V.

Lymph node mapping in 583 oral cavity cases by Pantvaidya et al. showed metastases to levels IV and V in 4.7% and 3.3%, respectively. They also concluded that level IIa is a guide for level V involvement [18]. Our analysis revealed similar results with level IV metastases in 2.2% and level V involvement in 4.4% of the cases. Overall, only 10.8% (9/83) of pN+ patients had positive lymph nodes in levels IV and V. This was true even in the tongue and lower alveolus subsites, which have a higher propensity for lymph nodal metastases.

Review for prognostic factors in OSCC by Massano et al. laid out tumor thickness, PNI, and stage of the disease as solid predictors of nodal metastases [19]. Likewise, predictive factors with statistical significance for pN+ in our study were DOI > 10 mm, PNI, and skin involvement at the primary site. Our analysis also showed that factors that predict nodal metastases, in general, do not predict metastases to levels IV and V.

Available evidence to support SND in node-positive oral cancers is level 2b (retrospective) at best. Based on these studies, presence of massive lymphadenopathy, multiple neck nodes, primary lesion in the hypopharynx, or oral cavity were contraindications for SND. Head and neck cancer patients with early T stage (T1, T2) and N1 disease can be considered for SND. Regional failure rates after SND in the node-positive neck varied from 4.3 to 16.9% [6, 13, 20, 21]. In the study of 401 neck dissections in 373 patients, Kokemueller et al. found regional recurrence rates of 20% with pN+ necks at 5-year follow-up [20]. Despite the comparable outcomes between SND and CND, one crucial point to note was the lack of uniform follow-up among SND arm studies. Available literature has been summarized in Table 5.

Key takeaways from this analysis were that all the cases with level IV metastases were of subsite tongue. Every case with level V involvement was of stage T4, and most of them were of subsite lower alveolus (4/6). The common predictive factor for level IV and V metastases was ENE/N3 disease. As per the eighth edition of AJCC, lesions with size > 4 cm and DOI > 10 mm have been upstaged as T4a lesions along with the previous definitions of bone, skin, and maxillary sinus mucosa invasion [22]. In our study, all the cases with DOI > 10 mm had primary lesions > 4 cm. Our analysis also showed skin involvement and DOI > 4 cm as predictive factors with cause-effect relation for nodal metastases. It implies that proceeding with a SND in a N3 disease in the neck may not be prudent in view of possible involvement of lower neck nodal levels. In addition, high chance of overall neck metastases exists with T4 primaries.

Our study's strength is that we analysed a specific subset of oral cancers (cT3/T4, cN+) in whom SND is still not practiced widely. The limitations include single institutional retrospective data, modest sample size, and lack of follow-up to know oncological outcomes. Currently, the exact predictive factors for level IV and V nodal metastases are not known. Extensive, multi-institutional studies are required to identify determinants of level IV and V metastases. Randomized control trials with follow-up on survival and functional outcomes are needed to expand the role of SND in cN+ oral cancers.

## 5. Conclusion

Our work supports the feasibility of considering SND, sparing levels IV and V in a subset of oral cancers with T3 stage with nodal stage N2 and below. However, an RCT involving a head-on comparison in terms of oncological outcomes and morbidity assessment between the current standard of comprehensive neck dissection versus an option of SND in a selected subset of patients as concluded from our study can provide a concrete and conclusive answer.

## Figures and Tables

**Table 1:** Clinical and pathological demographics.

		Number of patients *N* (%)
Age	<60	112 (83.6%)
	>60	22 (16.4%)

Sex	Male	119 (88.8%)
	Female	15 (11.2%)

Addiction	Smokeless tobacco	99 (73.9%)
	Smoking	65 (48.5%)
	Alcohol	60 (44.8%)

Comorbidities	CCI ≤ 2 no	62 (46.3%)
	CCI ≥ 3 yes	72 (53.7%)

Subsite	Buccal mucosa	70 (52.2%)
	Tongue	29 (21.6%)
	Upper alveolus	02 (1.5%)
	Lower alveolus	23 (17.2%)
	Mucosal lip	06 (4.5%)
	Hard palate	01 (0.7%)
	Retro molar trigone	03 (2.2%)

cN stage	1	35 (26.1%)
	2	77 (57.5%)
	3	22 (16.4%)

Crossing midline	Yes	33 (24.6%)
	No	101 (75.4%)

Skin excision	Yes	75 (56%)
	No	59 (44%)

pT	T3	45 (31.3%)
	T4	89 (66.4%)

pN stage	0	51 (38.1%)
	1	25 (18.7%)
	2	39 (29.1%)
	3	19 (14.2)

Grade	Well differentiated	60 (44.8%)
	Moderately differentiated	73 (54.5%)
	Poorly differentiated	01 (0.7%)

DOI	<10 mm	18 (13.4%)
	>10 mm	116 (86.6%)

PNI	Present	23 (17.2%)
	Absent	111 (82.8%)

LVI	Present	18 (13.4%)
	Absent	116 (86.6%)

ENE	Present	22 (16.4%)
	Absent	112 (83.6%)

Bone involvement	Present	46 (34.3%)
	Absent	88 (65.7%)

Skin involvement	Present	56 (41.8%)
	Absent	78 (58.2%)

CCI: Charlson comorbidity index; DOI: depth of invasion; PNI: perineural invasion; LVI: lymphovascular invasion; ENE: extranodal extension.

**Table 2:** Pathological lymph node distribution by T stage.

pT stage		pN *N* (%)	Ia *N* (%)	Ib *N* (%)	IIa *N* (%)	IIb *N* (%)	III *N* (%)	IV *N* (%)	V *N* (%)	Total
T3	N+	30 (66.7%)	5 (11.1%)	21 (46.7%)	20 (44.4%)	2 (4.4%)	7 (15.6%)	1 (2.2%)	0 (0%)	45
	N0	15 (33.3%)	40 (89.9%)	24 (53.3%)	25 (55.6%)	43 (95.6%)	38 (84.4%)	44 (97.8%)	45 (100%)	

T4	N+	53 (59.6%)	15 (16.9%)	35 (39.3%)	33 (37.1%)	5 (5.6%)	15 (16.9%)	2 (2.2%)	6 (6.7%)	89
	N0	36 (40.4%)	74 (83.1%)	54 (60.7%)	56 (62.9%)	84 (94.4%)	74 (83.1%)	87 (97.8%)	83 (93.3%)	

**Table 3:** Pathological lymph node distribution by N stage.

pN stage		Ia *N* (%)	Ib *N* (%)	IIa *N* (%)	IIb *N* (%)	III *N* (%)	IV *N* (%)	V *N* (%)	Total *N* (%)
N0		114 (85.1%)	78 (58.2%)	81 (60.4%)	127 (94.8%)	112 (83.6%)	131 (97.8%)	128 (95.5%)	51 (38.1%)

N+	N1	2 (1.5%)	9 (6.7%)	11 (8.2%)	1 (0.7%)	0 (0%)	0 (0%)	0 (0%)	25 (18.7%)
	N2	8 (6.0%)	31 (23.0%)	26 (19.4)	3 (2.2%)	8 (6.0%)	2 (1.5%)	2 (1.5%)	39 (29.0%)
	N3	10 (7.5%)	16 (11.9%)	16 (11.9%)	3 (2.2%)	14 (10.4%)	1 (0.7%)	4 (3%)	19 (14.2%)
	N+	20 (15%)	56 (41.8%)	53 (39.6%)	7 (5.2%)	22 (16.4%)	3 (2.2%)	6 (4.5%)	83 (61.9%)

**Table 4:** Association between clinicopathological factors and nodal metastases.

Variable	*p* value (for pN+)	*p* value (for level V pN+)	*p* value (for level IV pN+)
Age	0.95	0.24	0.14
Sex	0.87	0.37	0.53
Smokeless tobacco	0.27	0.13	0.29
Smoking	0.42	0.94	0.52
Alcohol	0.31	0.05	0.68
Multiple comorbidities	0.35	0.51	0.64
Subsite	0.26	0.08	0.08
Crossing midline	0.29	0.61	0.002
Skin excision	0.05	0.58	0.42
pT	0.42	0.07	0.99
Grade	0.68	0.34	0.74
DOI	0.007	0.32	0.49
PNI	0.001	0.28	0.42
LVI	0.01	0.14	0.49
ENE	—	0.001	0.01
Bone invasion	0.18	0.08	0.20
Skin invasion	<0.001	0.66	0.13

DOI: depth of invasion; PNI: perineural invasion; LVI: lymphovascular invasion; ENE: extranodal extension; pN+: pathological node positive.

**Table 5:** Previous studies on SND in node-positive head and neck cancers.

Author	Sample size	Site	Stage	Regional failure	Conclusion
Kowalski and Carvalho [21] (2002)	*n* = 164 SND in none	Oral cavity (100%)	T1 (3%), T2 (45.7%), T3 (28.7%), T4 (22.6%)	—	SND could be done in patients with cN+ at level 1
Andersen et al. [6] (2002)	*n* = 106 SND in 100%	Oral cavity (39.6%), oropharynx (34.9%), larynx (18.9%), hypopharynx (6.6%)	T1 (8.5%), T2 (26.4%), T3 (34.0%), T4 (30.2%)	4.3%	SND can be done in N+ head and neck cancers without massive adenopathy
Santos et al. [7] (2006)	*n* = 191 SND in 28/191 (14.6%)	Oral cavity (14.3%), oropharynx (3.5%), larynx (60.8%), hypopharynx (17.9%), nasopharynx (3.5%)	T1 (7.1%), T2 (7.1%), T3 (25%), T4 (60.8%)	(4/28) 14.2%	SND may be done in selected cases of T1, T2 with N1
Patel et al. [8] (2008)	*n* = 205 (232) SND in 72/232 (31%)	Oral cavity (32.6%), oropharynx (34.6%), larynx (10.2%), hypopharynx (22.4%)	T1-T2 (45.3%), T3-T4% (54.7%)	(19/205) 9.2%	SND can be done in selected N+ cases based on site (poor in hypopharynx), T stage (good in T1 & T2), and N stage (up to N1)
Shin et al. [9] (2013)	*n* = 92 SND in 20/92 (21.7%)	Oral cavity (100%)	T1 (13%), T2 (35.8%), T3 (13%), T4 (38%)	(3/20) 15%	SND combined with adjuvant therapy, survival rate comparable to CND in patients under cN2a OSCC
Givi et al. [10] (2011)	*n* = 108 SND in all	Oral cavity (71.3%), oropharynx (22.2%), larynx (5%), hypopharynx (1%), nasopharynx (1%)	T1 (14.8%), T2 (44.4%), T3 (9.3%), T4 (18.5%)	(7) 6.5%	SND can be done in selected N+ cases in the setting of MMT. Poor prognosis in oral cavity primaries, >2 neck nodes, and high T stage (T3 and T4)
Feng et al. [11] (2014)	*n* = 143 SND in 68/143 (47.5%)	Oral cavity (100%) (tongue & floor of mouth)	T1 (10.5%), T2 (19%), T3 (33%), T4 (37.3%)	(11/65) 16.9%	SND can be done in selected N+ cases
Iqbal et al. [12] (2014)	*n* = 219 SND in 100% cN+ in 61 (27.8%)	Oral cavity (100%)	T1 (44%), T2 (37%), T3 (6%), T4 (9%)	(8/61) 13%	SND can be done in N+ oral cancers
Shimura et al. [13] (2019)	*n* = 68 SND in 35/68 (51%)	Oral cavity (100%)	T1(18%), T2(47%) T3(9%), T4(26%)	(4/35) 11%	SND can be done in N+ limited to levels I and II, for up to 2 lymph nodes that are <3 cm
Kakei et al. [23] (2020)	*n* = 100 MND in all	Oral cavity (100%)	T1 (4%), T2 (49%), T3 (22%), T4 (25%)	—	Level V dissection can be excluded

SND: selective neck dissection; MND: modified neck dissection; CND: comprehensive neck dissection; MMT: multimodality treatment.

## Data Availability

The retrospective data used to support the findings of this study are restricted by the Institutional Ethics Committee of AIIMS, Rishikesh, in order to protect patient privacy. Data are available from Dr. Rajkumar K. Seenivasagam MS, MCh (Surgical Oncology), FRCS Ed, MFST Ed, FACS Sub-Dean (Planning), Associate Professor of Surgical Oncology, All India Institute of Medical Sciences Rishikesh International Surgical Advisor, the Royal College of Surgeons of Edinburgh for researchers who meet the criteria for access to confidential data.
